# 910. Evaluation of an Enzymatic Immunoassay System for Detection and Differentiation of Rat Hepatitis E Virus Infection in Humans

**DOI:** 10.1093/ofid/ofab466.1105

**Published:** 2021-12-04

**Authors:** Siddharth Sridhar, Jianwen Situ, Kelvin Hon Yin Lo, Jianpiao Cai

**Affiliations:** Department of Microbiology, The University of Hong Kong, Hong Kong

## Abstract

**Background:**

Previously, hepatitis E in humans was believed to be caused exclusively by species A variants of the *Orthohepevirus* genus (HEV-A) of the family *Hepeviridae*. However, we have previously demonstrated that *Orthohepevirus* species C (HEV-C), also known as rat hepatitis E virus, also causes hepatitis in humans. Due to high sequence divergence between HEV-A and HEV-C, serological tests based on HEV-A are often insensitive for HEV-C diagnosis. Therefore, we developed an enzymatic immunoassay (EIA) system for differentiating HEV-A and HEV-C antibody signatures in patient sera.

**Methods:**

HEV-A and HEV-C peptide homologs spanning the entire immunogenic E2s region of HEV ORF2 capsid protein were expressed in *E. coli*. These peptides, HEV-A4 p239 and HEV-C p241, form virus-like particles (figure 1). Both peptides were coated in separate 96-well plates. Sera obtained from patients with RT-PCR confirmed acute HEV-A infection (n = 54), HEV-C infection (n = 15), and uninfected HEV seronegative controls (n = 126) were tested in parallel in HEV-A4 p239 and HEV-C p241 EIAs for respective IgG antibodies. Sample optical densities (ODs) were divided by mean OD + 3SD of the seronegative controls to generate signal/noise (S/N) ratios. S/Ns marking positivity in either assay were determined by ROC analysis. An algorithm for assay interpretation was developed (table 1) and the performance of this algorithm was measured against the RT-PCR gold standard.

HEV-A4 p239 and HEV-C1 p241 virus like particles

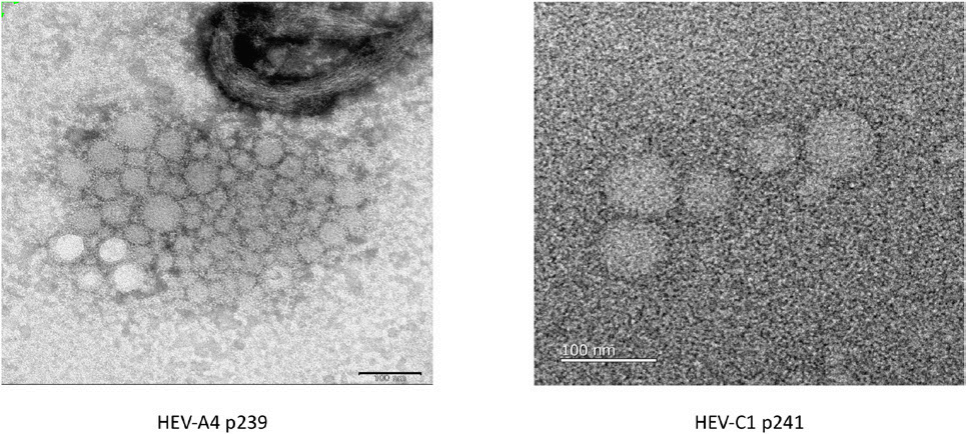

Transmission electron microscopy images of the two peptides used in this study

Diagnostic testing algorithm interpretation

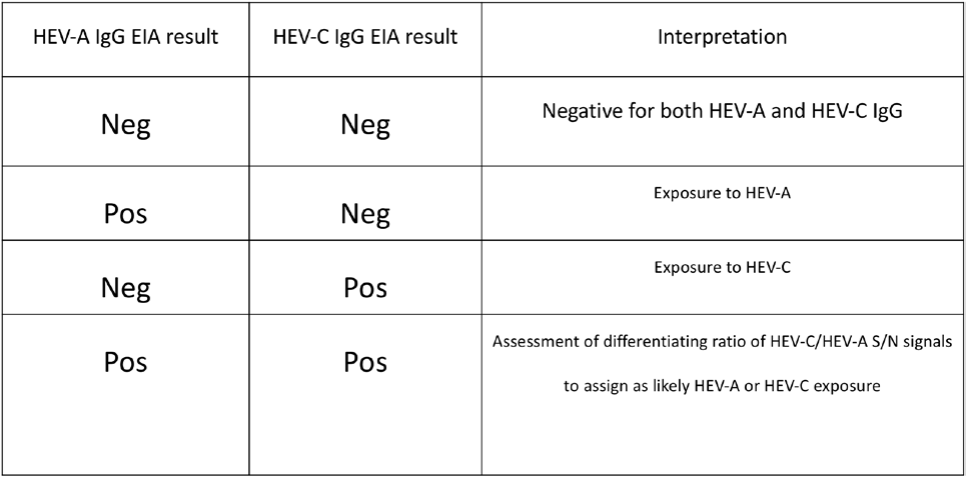

Interpretation of results of testing using parallel enzymatic immunoassays

**Results:**

Using cutoffs determined by ROC analysis (figure 2), HEV-A4 p239 and HEV-C p241 EIAs detected species-specific antibody responses well (sensitivity: 92.6% and 80% respectively) and were specific (92.9% and 98.3% respectively). The DC was 100% congruent with HEV-C RT-PCR and 88.9% congruent with HEV-A RT-PCR in RT-PCR positive samples. Incorporating all three cutoffs into the algorithm, we derived a 3×3 confusion matrix of RT-PCR sample assignation vs EIA algorithm classification (table 2). The Cohen’s κ value was 0.883 indicating excellent inter-rater reliability.

ROC analysis for determining S/N cutoffs

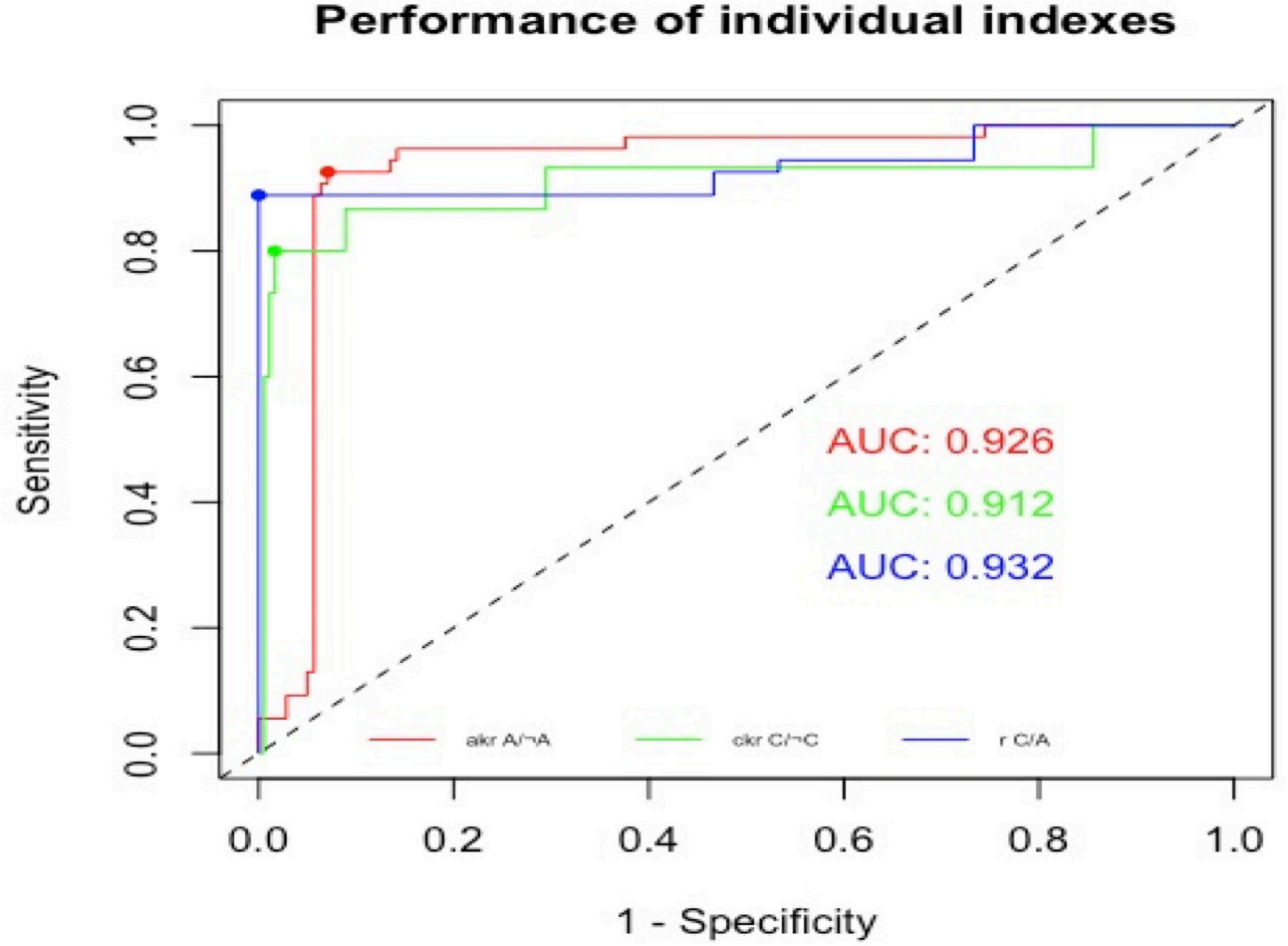

Curve for A/A represents analysis for HEV-A4 p239 EIA. Curve for C/¬C represents analysis for HEV-C p241 EIA. Curve for r(C/A) represents analysis for the differentiating ratio.

3×3 confusion matrix comparing sample assignations by RT-PCR vs. EIA algorithm

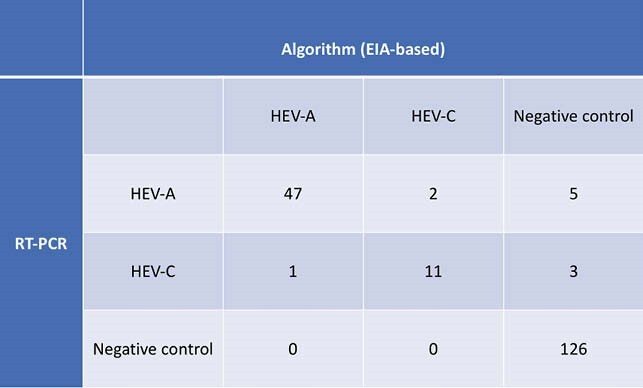

**Conclusion:**

A parallel EIA system accurately differentiated HEV-A and HEV-C serological signatures in acute patient sera. This method can now be applied to seroprevalence studies to determine seroprevalence of rat hepatitis E in human populations.

**Disclosures:**

**Siddharth Sridhar, FRCPath**, **Abbott** (Other Financial or Material Support, Speaker’s honoraria)

